# p38 MAPK-induced MDM2 degradation confers paclitaxel resistance through p53-mediated regulation of EGFR in human lung cancer cells

**DOI:** 10.18632/oncotarget.6945

**Published:** 2016-01-19

**Authors:** Shin-Hyung Park, Myeong-A Seong, Ho-Young Lee

**Affiliations:** ^1^ College of Pharmacy, Seoul National University, Seoul 08826, Republic of Korea

**Keywords:** paclitaxel resistance, epidermal growth factor receptor, p38 MAPK, p53, MDM2

## Abstract

Paclitaxel (PTX) is a chemotherapeutic agent that is used to treat a variety of cancers, including non-small cell lung cancer (NSCLC). However, the emergence of drug resistance limits the utility of PTX. This study determined the signaling pathway that contributes to PTX resistance. We first established PTX resistant cell lines (H460/R and 226B/R) using a dose-escalating maintenance of PTX. We found that p38 MAPK and epidermal growth factor receptor (EGFR) were constitutively activated in these cell lines. The inhibition of p38 MAPK activity by SB203580 treatment or the transfection of dominant-negative p38 MAPK sensitized both cell lines to PTX treatment. Erlotinib, an EGFR inhibitor, also increased PTX-induced apoptosis in PTX resistant cells, which suggests a role for p38 MAPK and EGFR in the development of PTX resistance. We demonstrated that p38 MAPK enhanced EGFR expression via the induction of the rapid degradation of mouse double-minute 2 homolog (MDM2) and the consequent stabilization of p53, a transcription factor of *EGFR*. These results suggest for the first time that the p38 MAPK/p53/EGFR axis is crucial for the facilitation of PTX resistance in NSCLCs. We also propose a mechanism for the role of the tumor-suppressor p53 in drug resistance. These results provide a foundation for the future development of potential therapeutic strategies to regulate the p38 MAPK/p53/EGFR pathway for the treatment of lung cancer patients with PTX resistance.

## INTRODUCTION

Paclitaxel (PTX) is a tubulin-disrupting agent that remains a first line chemotherapy in the management of advanced non-small cell lung cancer (NSCLC) [1]. However, the development of intrinsic or acquired resistance limits the utility of PTX [2, 3]. Therefore, investigations of the underlying mechanisms are needed to devise novel strategies to overcome chemoresistance.

Various mechanisms, including the overexpression of drug efflux pumps [4, 5], mutations in *TUBB* [6, 7], and aberrations in the molecular pathways of cell cycle control [8], confer PTX resistance. PTX sensitivity is dependent on molecules related to functional spindle assembly checkpoints [9]. p53 is a tumor suppressor protein that leads to cell growth arrest or apoptosis in response to DNA damage. As p53 is also implicated in the mitotic checkpoint [10], a hypothesis that p53 is an important determinant of cellular sensitivity to PTX has been suggested. For example, the activation of p53 promotes apoptosis in PTX resistant cancer cells, and the loss of functional p53 facilitates acquired resistance to PTX [11, 12]. In contrast, other researchers have demonstrated that the loss of p53 function sensitizes murine fibroblasts and cancer cells to PTX [13, 14], which leaves the controversial role of p53 in PTX resistance. These studies only focused on primary PTX resistance, Therefore, the role of p53 in acquired PTX resistance is largely unknown.

p38 mitogen-activated protein kinase (MAPK) is a stress-activated protein kinase (SAPK) that it is activated by a wide range of environmental stresses. It is most frequently associated with a tumor-suppressor function because it negatively regulates cell survival and proliferation [15]. PTX also induces apoptosis through a p38 MAPK-mediated pathway [16, 17]. In contrast, the role of p38 MAPK as a contributor to drug resistance was recently suggested. The inhibition of p38 MAPK diminished chemoresistance against drugs such as doxorubicin, daunorubicin, and vincristine by abrogation of the activity or expression of the P-glycoprotein (P-gp) protein [18–20]. A constitutive increase in phosphorylated p38 MAPK was found in drug-resistant cells. Notably, p38 MAPK also conferred PTX resistance to ovarian cancer cells, but the precise molecular mechanism has not been determined [12, 21]. These conflicting roles of p38 MAPK in PTX resistance suggest that the different downstream effectors that lead to the dual functions of p38 MAPK should be further elucidated.

Previous studies have demonstrated the mechanism of regulation of p53 stability. The most recognized player for mediating p53 protein degradation is mouse double-minute 2 homolog (MDM2). MDM2 serves as a typical E3 ubiquitin ligase of p53 and is also one of p53 target genes [22]. p38 MAPK-induced phosphorylation of p53 leads to its disassociation from MDM2 and consequent evasion of ubiquitin-proteasomal degradation [23]. p38 MAPK is also known to regulate MDM2 expression by unknown mechanism, suggesting the critical role of p38 MAPK in the modulation of MDM2 and p53 expression in a post-translational manner [24–27]. Although previous reports have demonstrated that MDM2 overexpression confers drug resistance through suppression of p53-mediated apoptosis [28, 29], its function as a negative regulator of chemoresistance remains unexplored.

This study determined the signaling molecules that contributed to PTX resistance. We found that p38 MAPK played a critical role in PTX resistance via the p53-mediated regulation of epidermal growth factor receptor (EGFR) expression. We propose a novel mechanism for p38 MAPK modulation of the EGFR pathway and the final facilitation of PTX resistance based on these observations. We also provide an explanation for the role of the tumor suppressor p53 in PTX resistance.

## RESULTS

### Establishment of PTX resistant lung cancer cell lines

**Figure 1:**
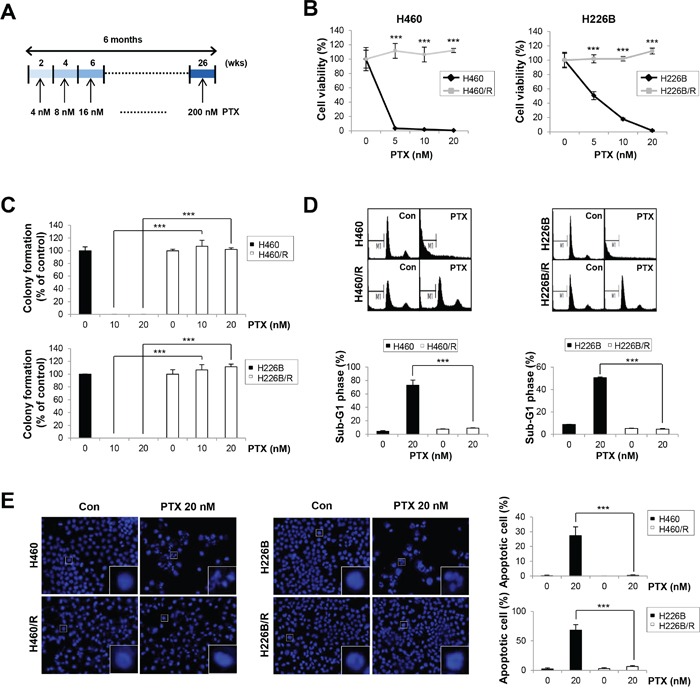
Establishment of PTX resistant cell lines **A.** Schedules for the creation of PTX resistant cell lines. Cells were treated with PTX at concentrations from 4 nM to 200 nM in a dose-escalating manner for 6 months. **B.** H460, H460/R, H226B, and H226B/R cells were treated with PTX in a dose-dependent manner for 72 h. Cell viability was evaluated using the trypan blue exclusion assay. **C.** Cells were grown for 10 days in medium containing 20 nM PTX. Anchorage-dependent colony formation was assessed by counting the individual colonies using Motic images plus 2.0 software. **D.** Cells were treated with 20 nM PTX for 72 h and incubated with PI solution for DNA content-based assessments of cell cycle distribution. The cell population in the sub-G1 phase was delineated as apoptotic cells. **E.** Fluorescence microscopy of DAPI-stained cells treated for 48 h with PTX (20 nM). Apoptotic cells were identified by highly condensed and fragmented nuclear DNA. The data are presented as the mean ± SD. The statistical significance was analyzed using Student's t-test (*** *P* < 0.001 vs. the respective control).

We treated H460 and 226B cells with 4 nM PTX, as a starting concentration, to generate PTX resistant sublines. The medium was changed to fresh medium that contained PTX every 3 days until the cells were confluent on the plates. The cells were incubated with gradually increasing PTX concentrations. This cycle was repeated for 6 months until the stable PTX resistant sublines, referred to as H460/R and H226B/R, were established (Figure 1A). We performed trypan blue exclusion and anchorage-dependent colony formation assays to determine whether PTX resistance was well established in these cell lines. The results demonstrated that the survival rates and colony forming ability of PTX resistant cells were significantly higher than those of the parental cells following PTX treatment (*P* < 0.001) (Figure 1, B and C). We conducted cell cycle analyses using flow cytometry to verify whether the resistant phenotypes were associated with reduced apoptosis. H460 and H226B cells exhibited a marked increase in sub-G1 phase populations compared with H460/R and 226B/R cells treated with PTX (*P* < 0.001) (Figure 1D). Similar results were obtained when apoptosis was monitored using DAPI staining. Apoptotic cells, identified by highly condensed and fragmented nuclear DNA, were increased in the parental cells following PTX treatment. In contrast, PTX-treated resistant cells exhibited a homogeneous staining of intact nuclei, similar to that of untreated control cells (Figure 1E). Taken together, these results demonstrated that H460/R and 226B/R cells acquired a PTX resistant phenotype.

### Identification of up-regulated signaling pathways in PTX resistant cells

**Figure 2:**
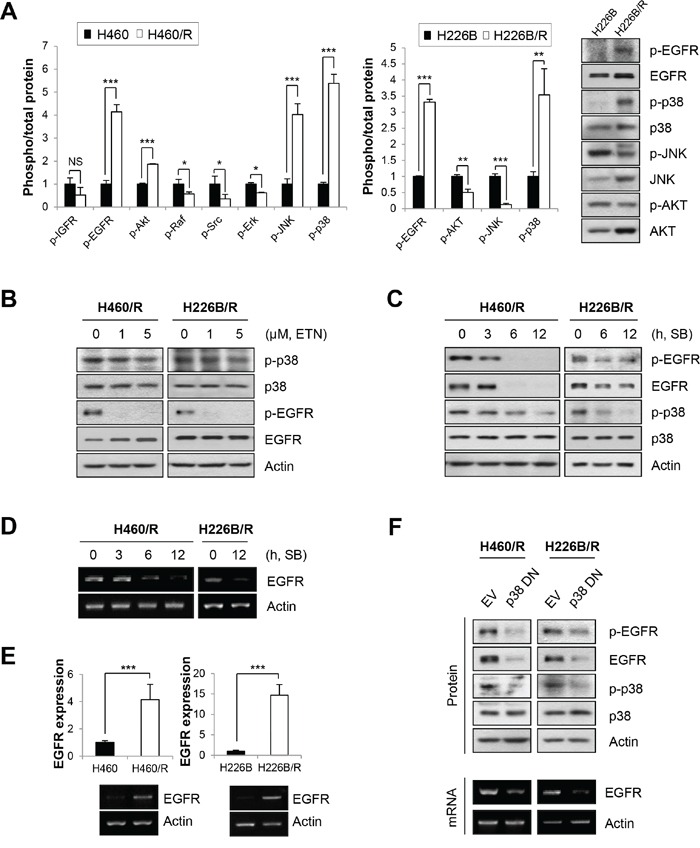
p-p38 MAPK and p-EGFR are up-regulated in PTX resistant cell lines **A.** The activities of signaling molecules related to cancer cell survival and the development of drug resistance were investigated in parental cells and PTX resistant cells. Activities were estimated by calculating the ratio between phosphorylation and total protein levels based on the band intensity of at least three immunoblots using ImageJ software (National Institutes of Health freeware). The right panel exhibits the representative immunoblots of H226B and H226B/R cells. The data are presented as the mean ± SD. The statistical significance was analyzed using Student's t-test (NS = not significant, * *P* < 0.05, ** *P* < 0.01, *** *P* < 0.001 vs. the respective control). **B.** H460/R and H226B/R cells were incubated with erlotinib (ETN) at the indicated concentrations for 24 h. The expressions of p-p38, p-EGFR and their total proteins were analyzed using western blot analysis. (C and D) H460/R and H226B/R cells were treated with SB203580 (SB; 50 μM) at the indicated time points. **C.** The expression of p-EGFR, p-p38 and their total proteins were analyzed using western blot analysis. **D.** The mRNA level of *EGFR* was determined using RT-PCR. **E.** The basal expression levels of *EGFR* mRNA between parental cells and PTX resistant cells were detected using RT-PCR (lower) and confirmed by quantitative RT-PCR (upper). The data are presented as the mean ± SD. The statistical significance was analyzed using Student's t-test (*** *P* < 0.001 vs. the respective control). **F.** H460/R and H226B/R cells were stably transfected with a dominant-negative mutant of p38 MAPK (p38 DN). Western blot analysis was performed to investigate the expression changes of p-EGFR and EGFR following the transfection of inactive p38 MAPK (upper). RT-PCR was also conducted to verify the influence of p38 MAPK on the expression of *EGFR* mRNA (lower).

We investigated constitutively activated signaling pathways in PTX resistant cells to identify the mechanisms that conferred PTX resistance. We focused on receptor tyrosine kinases (RTKs), including IGFR and EGFR, and their downstream signaling mediators, including Ras/Raf/MAPK and PI3K/AKT. These molecules are aberrantly activated due to overexpression or mutation in various human cancers, and they play crucial roles in cancer cell proliferation, anti-apoptotic behavior, and anticancer drug resistance [30–35]. Among these candidates, EGFR, AKT, JNK and p38 MAPK exhibited higher activities in H460/R cells compared with H460 cells (*P* < 0.001). Especially, EGFR and p38 MAPK were commonly activated in H226B/R and H460/R cells compared with their respective parental cells (Figure 2A). These results suggest that p38 MAPK and EGFR play an important role in PTX resistance.

MAPK is generally activated by signals that are transmitted by receptors located in the cell membrane. Therefore, we hypothesized that EGFR would be upstream of p38 MAPK. However, we found that the inhibition of EGFR by erlotinib hardly affected phosphorylated p38 (p-p38) levels, in contrast to our hypothesis (Figure 2B). Rather, an inhibitor of p38 MAPK, SB203580, reduced the expression of phosphorylated EGFR (p-EGFR) and total EGFR (t-EGFR) (Figure 2C). The mRNA expression of *EGFR* also decreased following SB203580 treatment, which suggests that the p38 MAPK-mediated regulation of EGFR expression occurs at the transcriptional level (Figure 2D).

We assessed the transcription level of *EGFR* in PTX resistant cells. Real-time PCR and RT-PCR analyses revealed elevated *EGFR* mRNA levels in H460/R and H226B/R cells compared with their corresponding parental cells (*P* < 0.001) (Figure 2E). Transfection with a dominant-negative mutant of p38 MAPK (p38 DN) reversed the up-regulation of *EGFR* mRNA and EGFR protein levels in PTX resistant cells (Figure 2F), which suggests that p38 MAPK functions upstream of EGFR. Collectively, these observations indicated that activation of the p38 MAPK/EGFR axis is associated with PTX resistance.

### Association of p38 MAPK with PTX resistance

**Figure 3:**
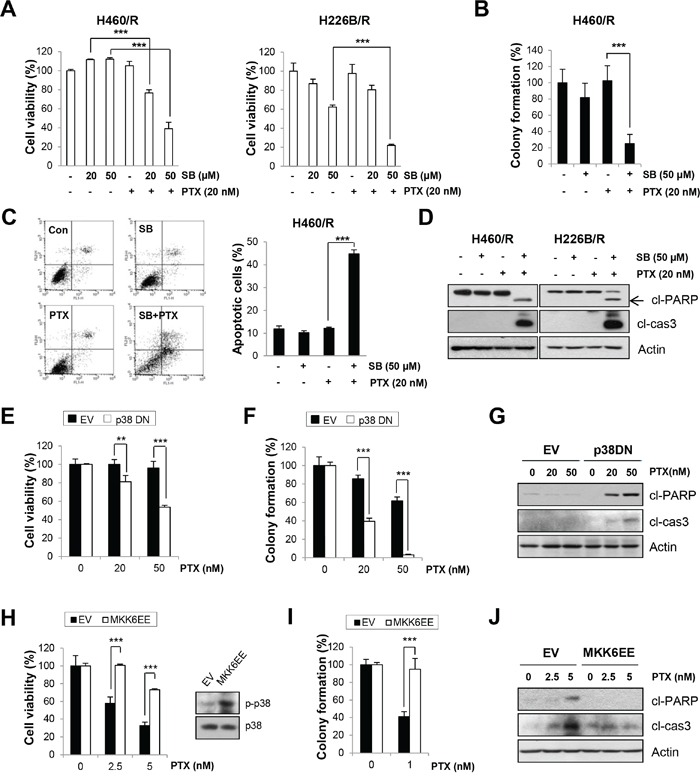
The activity of p38 MAPK determines PTX resistance **A.** H460/R and 226B/R cells were treated with PTX (20 nM) w/or w/o SB203580 (SB; 20 or 50 μM) for 72 h. Cell viability was determined by the MTT assay. **B.** For the anchorage-independent colony formation assay, a small number of H460/R cells was seeded in 24-well plates and co-treated with PTX (20 nM) and SB203580 (SB; 50 μM) for 10 days. The individual colonies were stained and counted, and the average number of colonies was calculated. **C.** H460/R cells were treated with PTX (20 nM) in the presence of SB203580 (SB; 50 μM) for 72 h, and annexin V/PI analysis was conducted. Annexin V-positive population was evaluated as apoptotic cells. **D.** H460/R and 226B/R cells were treated with PTX (20 nM) and SB203580 (SB; 50 μM) for 72 h and then harvested for western blot analysis. **E-G.** Cells stably transfected with empty (EV) or p38 DN-expression vectors were assessed with (E) the MTT assay to evaluate cell viability following PTX treatment (100 or 200 nM) for 72 h. (F) For the anchorage-dependent colony formation assay, a small number of cells was seeded in 12-well plates and challenged with PTX (20 or 50 nM) for 10 days. **G.** Western blot analysis was conducted following PTX treatment (50 or 100 nM) for 72 h to evaluate the influence of p38 MAPK on PTX resistance. **H-J.** Cells stably transfected with empty (EV) or MKK6EE-expression vectors were assessed with (H) the MTT assay following 72 h treatment with PTX (2.5 or 5 nM), (I) the anchorage-dependent colony formation assay after a 10-day treatment with PTX (1 nM), and (J) western blot analysis to detect the expression of apoptotic proteins following PTX treatment (2.5 or 5 nM) for 72 h. The data are presented as mean ± SD. The statistical significance was analyzed using Student's t-test (** *P* < 0.01, *** *P* < 0.001 vs. respective control).

We verified whether p38 MAPK activity was responsible for PTX resistance. SB203580 treatment sensitized H460/R and H226B/R cells to PTX, as shown in the MTT (Figure 3A) and soft agar colony-forming (Figure 3B) assays. Co-treatment with SB203580 and PTX also enhanced apoptosis, as evaluated by increase of Annexin V-positive populations and up-regulation of cleaved-PARP and cleaved-caspase-3 expressions, compared with single treatment with PTX (Figure 3, C and D). We used a genetic approach by introducing p38 DN into H460/R cells to exclude the effects of off-target activity of SB203580. Cells expressing p38 DN exhibited more significant decreases in cell viability and colony-forming ability compared with control cells following treatment with PTX (50 nM; *P* < 0.001) (Figure 3, E and F). PTX treatment also markedly increased the protein levels of cleaved PARP and cleaved caspase-3 compared with empty vector (EV)-transfected cells (Figure 3G). These results demonstrate that p38 MAPK plays a critical role in the generation of PTX resistance.

A constitutively active mutant of MAP kinase kinase 6 (MKK6EE), which directly phosphorylates p38 MAPK, was introduced in H460 parental cells to clarify whether p38 MAPK activity by itself was sufficient to create PTX resistance. We observed that transfection with MKK6EE rescued cell viability and colony-forming ability following PTX treatment (Figure 3, H and I). PTX-induced apoptosis, evaluated by cleaved PARP and cleaved caspase-3 expressions, was also decreased by MKK6EE transfection than mock transfection (Figure 3J), which indicates that p38 MAPK can confer PTX resistance.

### Effects of EGFR inhibition on PTX resistance

**Figure 4:**
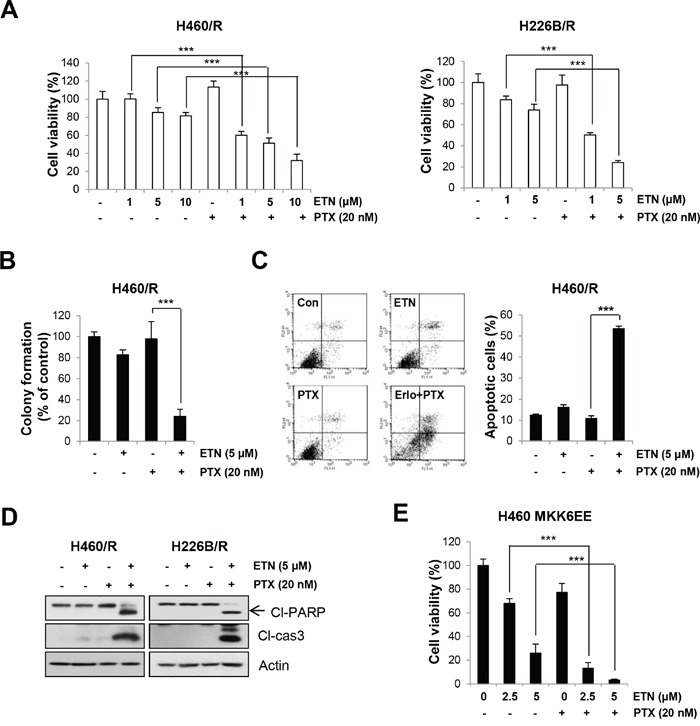
Inhibition of EGFR activity enhances the sensitivity to PTX **A.** H460/R and H226B/R cells were treated with erlotinib (ETN) in a dose-dependent manner in the presence of PTX (20 nM) for 72 h. Cell viability was determined by the MTT assay. **B.** For the anchorage-independent colony formation assay, a small number of H460/R cells were seeded in a 24-well plate and co-treated with PTX (20 nM) and erlotinib (ETN; 5 μM)) for 10 days. The individual colonies were stained and counted, and the average number of colonies was calculated. **C.** H460/R cells were treated with PTX (20 nM) in the presence of erlotinib (ETN or Erlo; 5 μM) for 72 h, and annexin V/PI analysis was conducted. Annexin V-positive population was evaluated as apoptotic cells. **D.** H460/R and H226B/R cells were treated with PTX (20 nM) and erlotinib (ETN; 5 μM) for 72 h and then harvested for western blot analysis. **E.** Cells stably transfected with the MKK6EE-expression vector were challenged with erlotinib (ETN; 2.5 or 5 μM) and PTX (1 nM) for 10 days for the anchorage-dependent colony forming assay. The individual colonies were stained and counted, and the average number of colonies was calculated. The data are presented as the mean ± SD. The statistical significance was analyzed using Student's t-test (*** *P* < 0.001 vs. the respective control).

Our data indicate that EGFR is downstream of p38 MAPK, and we postulated that targeting the EGFR pathway would be an effective strategy to overcome PTX resistance. Erlotinib treatment sensitized H460/R and H226B/R cells to PTX treatment. The combined treatment with PTX and erlotinib exhibited significant decrease in the cell viability and colony-forming ability than erlotinib single treatment in these two resistant sublines (*P* < 0.001) (Figure 4A and 4B). In addition, we observed that treatment with PTX alone did not cause apoptotic cell death, whereas combinatorial treatment with erlotinib markedly restored PTX-induced apoptosis, as determined by increases in annexin V-positive cell population (*P* < 0.001), in H460/R cells (Figure 4C). The expression of cleaved-PARP and cleaved-caspase-3 was also increased by co-treatment with PTX and erlotinib in PTX resistant cells (Figure 4D).

To verify whether the p38 MAPK-induced PTX resistance is mediated by the EGFR signaling pathway, we examined whether erlotinib treatment sensitizes MKK6EE-overexpressing cells to PTX. The results clearly showed that the combined treatment of PTX with erlotinib markedly reduced the colony formation in MKK6EE cells compared with erlotinib single treatment (*P* < 0.001) (Figure 4E). These observations suggest that p38 MAPK confers PTX resistance through modulation of the EGFR pathway.

To investigate whether p38 MAPK regulates EGFR pathway in parental cells as well, we examined whether blockade of p38 MAPK can inhibit the expression of p-EGFR and t-EGFR in H460 and H226B cells. As shown in Supplementary Figure 1A, SB203580 slightly reduced (H460 cells) or even increased (H226B cells) p-EGFR expression without alteration of total EGFR (t-EGFR) expression levels. In addition, neither SB203580 nor erlotinib enhanced the effect of PTX in these naïve cell lines (Supplementary Figure 1, B and C). These results suggest that regulation of EGFR by p38 MAPK suppression sensitizes only NSCLC cells carrying acquired PTX resistance, but not parental cells, to the drug treatment.

Next, we assessed whether inhibition of AKT or JNK could sensitize H460/R cells to PTX treatment, because they were up-regulated in H460/R cells compared with H460 cells (*P* < 0.001) (Figure 2A). We observed that neither LY294002, a PI3K/Akt inhibitor, nor SP600125, a JNK inhibitor, sensitized H460/R cells to PTX treatment (Supplementary Figure 2, A and B). These results clearly suggest that activation of AKT and JNK in H460/R cells is not associated with PTX resistance.

As previous reports showed that p38 MAPK is responsible for drug resistance through regulation of multidrug resistance 1 (MDR1) gene [18, 19], we examined whether p38 MAPK-mediated development of PTX resistance is also related with up-regulation of drug efflux pumps. First, we investigated whether MDR genes are up-regulated in PTX resistant cells. We particularly focused on MDR1/ABCB1 and ABCB4 genes according to the previous report demonstrating increase in these two genes in the most in H460-PTX resistant cells compared with parental cells [36]. Through real-time quantitative reverse transcriptase PCR (qRT-PCR), we observed that both *ABCB1* and *ABCB4* increased in H460/R and H226B/R cells compared with their respective parental cells (*P* < 0.001) (Supplementary Figure 3, A and B). These results are in agreement with the previous reports which indicated the overexpression of drug efflux pumps as a mechanism of PTX resistance [4, 5]. To investigate whether these genes are regulated by p38 MAPK as reported in the earlier studies [18, 19], we treated the PTX resistant cells with SB203580 and examined the changes in mRNA expression of ABCB1 and ABCB4 using qRT-PCR. Contrary to the previous reports, our results showed that ABCB1 gene was even increased by SB203580 treatment in H460/R and H226B/R cells. The expression of ABCB4 gene was not affected by SB203580 treatment in H226B/R cells. Even though SB203580 treatment reduced the expression of ABCB4 in H460/R cells, the extent of fold increase in ABCB4 was quite low compared with that of ABCB1, suggesting that their contribution to PTX resistance would be marginal (Supplementary Figure 3, A and B). Collectively, these results suggest that p38 MAPK/p53-mediated PTX resistance is not related to the regulation of MDR genes, highlighting our findings as an additional novel mechanism of PTX resistance.

### Involvement of p53 in the p38 MAPK-mediated regulation of EGFR expression

**Figure 5:**
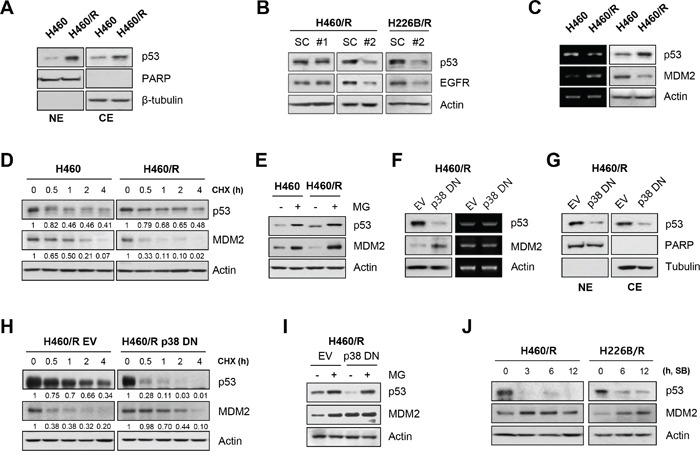
p38 MAPK regulates EGFR expression through the rapid degradation of MDM2 and increase in p53 stability **A.** The nuclear translocation of p53 was examined using nuclear/cytosol extraction in H460 and H460/R cells. PARP and β-tubulin were used as markers of nuclear and cytosolic fractions, respectively. **B.** H460/R and H226B/R cells were transfected with scrambled (SC) or TP53 siRNAs (#1 or #2). At 48 h post-transfection, the cells were collected and assessed for western blot analysis to investigate the influence of p53 on the expression of EGFR. **C.** The basal expression level of p53 and MDM2 in H460 and H460/R cells was investigated using western blot (left) and RT-PCR (right) analyses. **D.** Cycloheximide (CHX) was added to the medium to stop protein translation. Cells were taken at the indicated time-points, and the protein turnover of p53 and MDM2 was determined using western blot analysis. The relative abundance of p53 and MDM2 compared with the untreated controls was calculated based on band intensity using ImageJ software. **E.** The cells were treated with MG-132 (MG; 10 μM) for 4 h, and the protein levels of p53 and MDM2 were determined using western blot analysis. **F-I.** H460/R cells were stably transfected with empty (EV) or p38 DN expression vectors. **F.** The protein level (left) and mRNA expression (right) of p53 and MDM2, **G.** the nuclear localization of p53, **H.** the protein turnover of p53 and MDM2 following CHX treatment, and **I.** the recovery of the amount of p53 and MDM2 protein following MG-132 treatment were assessed. **J.** H460/R and H226B/R cells were treated with SB203580 (SB; 50 μM) in a time-dependent manner. The expression changes in p53 and MDM2 were detected using western blot analysis.

We investigated the transcription factors for *EGFR* gene expression to determine the mechanisms of the p38 MAPK regulation of EGFR expression. We focused on p53 as a target factor based on previous studies for the following reasons: (i) wild-type *p53* transcriptionally regulates the *EGFR* gene via direct DNA binding [37–39]; (ii) p38 MAPK enhances p53 protein stability and transcriptional activity via the phosphorylation of N-terminal residues [40, 41]; (iii) co-overexpression of p53 protein and EGFR correlated with poor prognosis and advanced pathological stage in papillary thyroid carcinomas and NSCLC [42, 43]; and (iv) the p38 MAPK/p53/p21 signaling axis confers primary resistance to PTX in prostate cancer cells [44]. We investigated whether p53 mediated the p38 MAPK-induced EGFR expression. We assessed the nuclear localization of p53 in H460/R cells. Western blot analysis demonstrated that nuclear and cytosolic p53 expression was greater in H460/R cells than in H460 cells (Figure 5A). To validate the role of p53 in EGFR expression, we analyzed EGFR expression in H460R and H226B/R cells, in which p53 expression was knocked-down by siRNA against *TP53*. Silencing of the *TP53* gene decreased EGFR expression. One siRNA (siTP53 #1), which failed to silence *p53* expression, did not influence EGFR expression (Figure 5B). Notably, p53 protein levels were elevated, but *TP53* mRNA levels were slightly lower in H460/R cells than in H460 cells (Figure 5C). As it is reported that p38 MAPK controls the stability of p53 [23, 40], we hypothesized that p38 MAPK would mediate p53 stabilization. We analyzed the half-life of the p53 protein and found that the half-life of p53 in H460/R cells was longer than in H460 cells (Figure 5D). Next, we assessed MDM2 expression in PTX resistant cells. *MDM2* mRNA levels were greater in H460/R cells than in H460 cells. However, MDM2 protein levels were greater in H460 cells than in H460/R cells (Figure 5C). Moreover, the half-life of MDM2 in H460/R cells was shorter than that in H460 cells (Figure 5D). MDM2 expression in H460/R cells was restored following treatment with the proteasome inhibitor MG-132, which indicates a ubiquitin-proteasomal pathway-mediated degradation of the MDM2 protein in H460/R cells (Figure 5E). These findings suggested that p53 and MDM2 proteins are under the post-translational regulation that occurs in H460/R cells.

**Figure 6:**
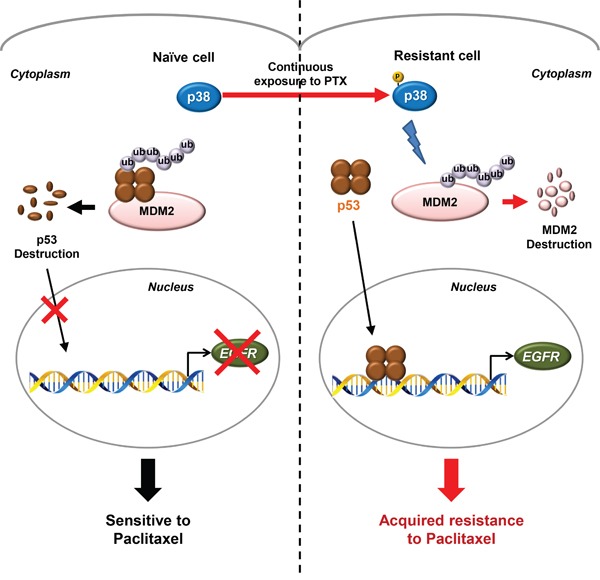
A schematic model of the p38 MAPK/p53/EGFR pathway that leads to PTX resistance Schematic diagram for the proposed model of the PTX resistance in NSCLC cells. Continuous exposure of cancer cells to PTX activates a stress-induced kinase p38 MAPK to develop PTX resistance. In chemo-naïve cells, p53 is generally degraded by its E3 ligase, MDM2, conferring the high sensitivity to PTX. However, the constitutively activated p38 MAPK in PTX-resistant cells induces the rapid degradation of MDM2 through the classical ubiquitin-proteasome pathway and the consequent stabilization of p53. The stabilized p53 translocates to the nucleus and regulates the expression of its target genes, including EGFR, which finally leads to the generation of acquired resistance to PTX.

We assessed whether p38 MAPK was involved in the post-translational regulation of p53 and MDM2 proteins in H460/R cells. H460/R cells, in which p38 MAPK was inactivated by transfection with dominant-negative p38 MAPK (p38 DN), exhibited decreased p53 protein and increased MDM2 protein levels and no changes in their mRNA expression levels (Figure 5F). The inactivation of p38 MAPK also decreased nuclear and cytosolic p53 protein levels (Figure 5G). p38 MAPK inactivation decreased p53 half-life but prolonged MDM2 half-life, which is consistent with our expectations (Figure 5H). In addition, MG-132 treatment thoroughly recovered the reduction of p53 protein expression (Figure 5I). These results indicated that the regulation of p53 stability by p38 MAPK was dependent on the MDM2-mediated ubiquitin-proteasome pathway. Application of the specific inhibitor of p38 MAPK SB203580 suppressed p53 expression but induced a simultaneous up-regulation of the MDM2 protein in H460/R and H226B/R cells (Figure 5J). Collectively, these data suggest that p38 MAPK protects p53 from the ubiquitin-proteasomal degradation via induction of the rapid destruction of MDM2, which ultimately leads to the up-regulation of its target genes, including *EGFR* (summarized in Figure 6).

## DISCUSSION

The present study demonstrated novel insight into the mechanism underlying acquired resistance to PTX. We identified the p38 MAPK/p53/EGFR axis as a pivotal signaling pathway that confers PTX resistance. p38 MAPK functions as a tumor suppressor, and we provided evidence that p38 MAPK contributed to chemoresistance via regulation of the EGFR pathway in a p53-dependent mechanism. One of the most significant advancements of this study is that the novel bidirectional modulation of MDM2 and p53 mediated by p38 MAPK is involved in the development of PTX resistance. We suggest that cells exposed to continuous stress utilize p38 MAPK and p53 to repair the accumulated cellular damage.

The role of the EGFR family in the emergence of PTX resistance was suggested in a series of studies: (i) gefitinib and lapatinib reversed PTX resistance in prostate, breast, and ovarian cancer cells via the direct inhibition of P-gp and drug efflux [45–47]; (ii) the expression of constitutively active EGFR and HER2 induces PTX resistance via increases in class IVa and IVb β-tubulin [48]; (iii) the overexpression of ErbB2 inhibits PTX-induced apoptosis via p21-dependent mechanisms [49, 50]; and (iv) ErbB3 contributes to PTX resistance via PI3K/Akt-mediated up-regulation of survivin [51]. Strategies to block the EGFR family enhanced the response to PTX in clinical trials. For example, trastuzumab significantly improved the response of breast cancer patients to PTX [52]. Our results also demonstrated that the EGFR pathway was activated in NSCLC cells sublines with acquired PTX resistance and that the PTX resistance was dramatically reduced when erlotinib was treated in combination with PTX. On the contrary, PTX plus erlotinib combination exhibited no synergy in parental cells. A phase III trial also demonstrated that combination therapy of gefitinib and PTX in chemotherapy-naïve patients with advanced NSCLC revealed no clinical benefit compared with standard chemotherapy alone [53]. These results highlight the importance of EGFR in the development of PTX resistance and suggest that EGFR expression is a critical issue to determine the sensitivity to PTX-based therapy.

In the current study, we found that the activation of p38 MAPK was related with the EGFR expression. The role of p38 MAPK in resistance to various chemotherapeutic drugs including PTX has been suggested in several studies [12, 18–21]. The present study demonstrated the following results: 1) p38 MAPK activation and transcriptional increases in *EGFR* expression in PTX resistant cells; 2) reduced *EGFR* transcription following treatment with SB203580 or transfection with p38 DN; and 3) significant attenuation of MKK6EE-induced PTX resistance by erlotinib treatment. These results clearly indicate that p38 MAPK induced PTX resistance via a transcriptional increase in *EGFR* expression. Several studies demonstrated that stress-induced activation of p38 MAPK enhances the cytotoxic effect of chemotherapy by transphosphorylation of EGFR at multiple serine and threonine residues and subsequent receptor internalization [54]. In contrast, our results demonstrated that p38 MAPK induced PTX resistance by phosphorylation of EGFR at tyrosine 1068, an autophosphorylation site. Mathay et al. reported that oxidative stress-induced p38 MAPK activation increased heparin-binding (HB)-EGF expression [55]. Ectodomain shedding of EGFR ligands including transforming growth factor-alpha (TGF-α) and pro-HB-EGF following p38 MAPK activation was also reported [56, 57]. These ligands resulted in subsequent EGFR activation and evasion of chemotherapeutic agent-induced apoptosis in tumor cells [57]. Thus, the modulation of EGFR ligand bioavailability is another possible mechanism through which p38 MAPK mediates EGFR pathway activation. Taken together, these findings suggest the importance of the p38 MAPK/EGFR pathway in chemotherapy tolerance and the development of drug resistance.

We elucidated the mechanisms underlying the p38 MAPK-mediated transcriptional increase in *EGFR* expression and found that p53 is the transcription factor for EGFR expression. Conflicting roles of p53 in PTX resistance were suggested in several studies. Most studies describe p53 as a mediator of PTX-induced apoptosis. However, some research suggests that the function of p53 in response to PTX is cell type-dependent. Notably, p38 MAPK and p53 were up-regulated in response to cellular damage, and cells that survived the prolonged treatment with PTX developed PTX resistance via the activation of stress-dependent pathways, including p38 MAPK and p53, to repair the accumulated cellular damage. Several studies suggested a mechanism for the p38 MAPK-based regulation of p53 expression. The most recognized mechanism is p38 MAPK-mediated phosphorylation of p53 at N-terminal serine residues, which results in disassociation from MDM2 and consequent evasion of MDM2-mediated ubiquitin-proteasomal degradation [23]. p38 MAPK is also associated with growth arrest and DNA damage-inducible 45α (GADD45α) proteins, and it promotes their interaction with p53, which further increases p53 stability [41, 58]. We found a rapid degradation of MDM2 and enhanced p53 stability in PTX resistant cells. This phenomenon was completely reversed by p38 MAPK inhibition, which suggests that p38 MAPK controlled the stability of MDM2 and p53 (Figure 5). Several studies reported the p38 MAPK-mediated regulation of MDM2 [24–27], but most of these studies provided no explanation of the underlying mechanism. Notably, Elias et al. reported that p38 MAPK markedly decreased steady-state levels of MDM2 through an indirect mechanism [27]. It seems that prolonged cellular stress induced by PTX treatment activates p38 MAPK stress kinase, which regulates p53 and MDM2. Lu et al. also demonstrated that docetaxel treatment reduced MDM2 expression and increased p53 stability, which is consistent with our results [44].

The current study did not address how p38 MAPK mediates the degradation of MDM2. It is likely that p38 MAPK directly phosphorylates MDM2, which leads to its degradation through the proteasome pathway. Alternatively, p38 MAPK may interact with other molecules to regulate MDM2 stability. DNA-dependent protein kinase (DNA-PK), ataxia-telangiectasia mutated (ATM) kinase, AKT, and cyclin-dependent kinase (CDK) are implicated in MDM2 phosphorylation which induces autoubiquitination of MDM2 [26]. Notably, ATM kinase is involved in the cellular response to genotoxic stress, and it exhibits reciprocal effects on MDM2 and p53 like p38 MAPK [26]. These results suggest that p38 MAPK might crosstalk with these molecules. We did not assess whether p38 MAPK directly phosphorylates p53 in PTX resistant cells, but our findings suggest that the p38 MAPK-mediated degradation of MDM2 plays a critical role in p53 stabilization.

In conclusion, we propose a paradoxical scenario in which the tumor-suppressor p53 confers PTX resistance. We suggest that the prolonged stress associated with PTX treatment stimulates the p38 MAPK/p53 network and induces *EGFR* transcription, which activates the EGFR pathway and PTX resistance in NSCLC. Our results also demonstrated significantly enhanced antitumor activities by co-treatment with SB203580 and PTX in NSCLC cells with acquired PTX resistance. These results suggest that targeting p38 MAPK, p53, or EGFR could be a therapeutic strategy to overcome PTX resistance. In support of our suggestion, a recent clinical trial demonstrates that the combinatorial treatment with an EGFR inhibitor and PTX improves the response of breast cancer patients to PTX [52]. In addition, development of various p38 MAPK inhibitors as anticancer therapeutics is ongoing [59], and a recent study demonstrates that a p38α inhibitor BIRB 796 enhances antitumor effects of anticancer therapies for multiple myeloma such as bortezomib and dexamethasone [60]. Further studies are warranted to evaluate the effectiveness of these combinatorial strategies in additional preclinical and clinical settings.

## MATERIALS AND METHODS

### Chemicals and reagents

PTX was purchased from Tocris (Minneapolis, MN, USA) and dissolved in dimethyl sulfoxide (DMSO, Sigma-Aldrich, St. Louis, MO, USA) to give the 100 mM stock solution. The PTX stock solution was stored at −70°C. SB203580, LY294002 and SP600125 were purchased from Millipore (Billerica, MA, USA), and erlotinib was purchased from LC Laboratories (Woburn, MA, USA). We purchased trypan blue from WelGENE (Daegu, Korea), and MTT [3-(4,5-dimethylthiazol-2-yl)-2,5-diphenyltetrazolium bromide] from MP Biomedicals, LLC (Illkirch, France). 4,6-dianmidino-2-phenylindole (DAPI) and propidium iodide (PI) were purchased from Sigma-Aldrich. Cycloheximide (CHX) was purchased from EMD Biosciences (San Diego, CA, USA), and MG-132 was purchased from Tocris. p53 siRNA and scrambled siRNA were synthesized from Genepharma (Shanghai, China), and Lipofectamine 2000 was purchased from Invitrogen (Carlsbad, CA, USA). Primary antibodies against p-p38 MAPK, p38 MAPK, p-JNK, JNK, AKT, and actin were purchased from Santa Cruz Biotechnology (Santa Cruz, CA, USA), and the other primary antibodies were purchased from Cell Signaling Technology (Beverly, MA, USA). Second antibodies were purchased from Santa Cruz Biotechnology.

### Cell culture

H460 cells were purchased from the American Type Culture Collection (ATCC; Manassas, VA, USA). H226B cells were kindly provided by Jack A. Roth (MD Anderson Cancer Center, Houston, TX, USA). Cells were grown at 37°C in a humidified incubator under 5% CO_2_ in RPMI 1640 supplemented with 10% fetal bovine serum (FBS) and antibiotics (all from WelGENE). Cells were sub-cultured every 2-3 days at approximately 80–90% confluency.

### Trypan blue exclusion assay

Cells were seeded in 12-well plastic culture dishes at a density of 1×10^4^ cells per well and incubated overnight. The medium was then replaced with complete media containing different concentrations of PTX. After 3 days, cells were collected and diluted 1:1 using a 0.4% trypan blue solution. As non-viable cells are stained as blue color, the number of viable cells was evaluated by counting unstained cells using a hemocytometer.

### MTT assay

Cells were seeded onto 96-well plates at a density of 3×10^3^ cells per well, and allowed to attach overnight. Cells were treated with different concentrations of PTX with or without erlotinib or SB203580 and incubated for 3 days. The MTT solution (final 0.4 mg/ml) was added and incubated for 3 h. The media were aspirated and 100 μl of DMSO were added to each well to dissolve the formazan product. After shaking for 30 min, the absorbance was measured at 570 nm using a microplate reader.

### Anchorage-dependent and -independent colony formation assay

The anchorage-dependent colony formation assay was conducted by seeding cells onto 12-well plates at a low density (2×10^2^ cells per well). Cells were treated with different concentrations of PTX with or without erlotinib and incubated for 10-14 days at 37°C in a humidified atmosphere with 5% CO_2_ until visable colonies were formed. During the period, the medium was changed every 3 days. The colonies were fixed with 100% methanol for 5 min and stained with hematoxylin for 30 min at room temperature. After washing several times with distilled water (DW), the number of visable colonies was counted.

For the anchorage-independent colony formation assay, 4% low-melting agarose (Lonza, Rockland, ME) solution was prepared in phosphate-buffered saline (PBS) as a stock solution. 1 ml of 1% bottom agar solution was added to each well in a 24-well cell culture plate and left to solidify at room temperature. Then 0.5-1×10^3^ cells were resuspended in 0.5 ml of top agar solution (final 0.4%) and plated. The plate was kept at room temperature until the top agar solidified and then incubated for 10-14 days in the growth medium containing different concentrations of PTX with or without erlotinib or SB203580. The medium was changed every 3 days during this period. The colonies were stained with the MTT solution (final 0.5 mg/ml) for 2 h at 37°C. After the medium containing MTT solution was changed with PBS, the colonies were photographed, and the number of visable colonies was counted.

### Flow cytometry analysis

For measurement of cell population in the sub-G1 phase, cells were seeded in 60-mm culture dish at a density of 5×10^5^ cells per dish. After overnight incubation, cells were treated with different concentrations of PTX for 72 h. Adherent and floating cells were collected, washed twice with PBS, and fixed with cold 80% ethanol for more than 1 h at 4°C. After centrifugation at 8,000 rpm for 3 min, cells were resuspended in 500 μl of PI/PBS staining solution which contains 450 μl of PBS, 50 μl of 0.5 mg/ml PI, and 1.5 μl of 10 mg/ml DNase-free RNase A (Sigma) to stain DNA, and then incubated for 30 min at room temperature. Flow cytometric analyses were performed using a flow cytometer (FACS Caliber, Becton Dickinson), and the relative DNA content in each phase of the cell cycle was determined by CellQuest software. The population in the sub-G1 phase was determined as apoptotic cells.

For Annexin V/PI double staining, cells were seeded onto 6-well plate at a density of 1.5×10^5^ cells per well. Then cells were challenged with PTX and erlotinib or SB203580 for 72 h. Apoptotic cells were quantitatively identified with the Annexin V-FITC Apoptosis Detection Kit I (BD Biosciences PharMingen, San Diego, CA, USA), following protocols provided by the manufacturer.

### Nuclear staining with DAPI

3×10^5^ cells were seeded onto cover glass in 6-well plate and treated with PTX the next day. After 48 h, cells were harvested, washed once with PBS, and then fixed with 3.7% paraformaldehyde (Sigma) in PBS for 30 min at room temperature. The fixed cells were washed three times with PBS, and stained with DAPI solution (2.5 μg/ml) for 20 min at room temperature. Cells were washed twice with PBS and mounted with a mounting solution (Vector Laboratories, Burlingame, CA, USA). The morphologic changes in the nucleus were analyzed by Nuance fluorescence microscope (Perkin-Elmer, Waltham, MA).

### Western blotting analysis

To prepare whole cell lysate, cells were lysed with ice-cold lysis buffer [1% NP-40, 0.25% sodium deoxycholate, 1 mM EDTA, 150 mM NaCl, 50 mM Tris–HCl (pH 7.4), 1% Triton X-100, 10% Glycerol] including a complete protease inhibitor cocktail tablet (Roche diagnostics, Mannheim, Germany) and phosphatase inhibitors (1 mM Na3VO4, 100 mM NaF, and 10 mM NaPP). After centrifugation at 13,000 rpm at 4°C for 30 min, the supernatants were collected and protein concentration was determined using a biochinconinic acid (BCA) protein assay kit (Pierce Biotechnology, Rockford, IL, USA) as described in the manufacturer's protocol. Equivalent amounts of protein were resolved by sodium dodecyl sulfate (SDS)–polyacrylamide gels and transferred to a polyvinyl difluoride (PVDF) membrane. After the membrane was blocked with 3% bovine serum albumin (BSA, GenDEPOT, Barker, TX, USA) in TBST [Tris-buffered saline (TBS) containing 0.1% Tween 20] for 1 h at room temperature, the membrane was incubated with primary antibody at the appropriate dilution in 3% BSA overnight at 4°C. The membrane was then washed multiple times with TBST and incubated with the appropriate horseradish peroxidase-conjugated secondary antibody for 1 h at room temperature. The protein–antibody complexes were detected by SuperSignal West Pico Chemiluminescent Substrate (Thermo Scientific Pierce, Rockford, IL, USA) according to the manufacturer's recommended protocol.

### RNA extraction and quantitative RT-PCR

Cells subjected to appropriate treatments were harvested and total RNA was extracted using TRIzol reagent (Invitrogen) according to the manufacturer's protocol. First strand cDNA was synthesized with the PrimeScript RT reagent Kit (TaKaRa, Dalian, China) using 1 μg of total RNA according to manufacturer's protocol. RT-PCR was performed to amplify genes using a cDNA template corresponding to gene-specific primer sets. The primer sequences used are as follows. EGFR: forward, 5′-TGG AGC TAC GGG GTG ACC GT-3′, reverse, 5′-GGT TCA GAG GCT GAT TGT GAT-3′, TP53: forward, 5′-CTG AAC AAG TTG GCC TGC AC-3′, reverse, 5′-GAA ATC CTC CAG GGT GTG GG-3′, MDM2: forward, 5′-ATG GTG AGG AGC AGG CAA AT-3′, reverse, 5′-GAT TCG ATG GCG TCC CTG TA-3′, ACTIN: forward, 5′-ACT ACC TCA TGA AGA TC-3′, reverse, 5′-GAT CCA CAT CTG CTG GAA-3′. PCR was performed in a total reaction volume of 20 μl that contained 1 μl of cDNA solution, 0.2 μM sense and antisense primers, and 10 μl of 2x MyTaq Red Mix (Bioline, Sydney, Australia). The RT-PCR exponential phase was determined on 20-32 cycles to allow quantitative comparisons among the cDNAs amplified from identical reactions. The amplification products were resolved on a 1.5% agarose gel, stained with RedSafe^TM^ nucleic acid staining solution (Intron Biotechnology, Seongnam-si, Korea), and visualized by Gel Doc image analysis system (Bio-Rad, Hercules, CA, USA), and photographed. Quantitative RT-PCR assays to detect mRNA expression were conducted using SYBR Premix Taq (TaKaRa) with actin as an internal control. The primer sequences used are as follows. EGFR : forward, 5′-CCT GGT CTG GAA GTA CGC AG-3′, reverse, 5′-CTT CGC ATG AAG AGG CCG AT-3′, ABCB1 : forward, 5′-GGA AAG TGC TGC TTG ATG GC-3′, reverse, 5′-AGG CAT GTA TGT TGG CCT CC-3′, ABCB4 : forward, 5′-GGG GAC AGT GTT TGT GGA CT-3′, reverse, 5′-TAC AAC CCG GCT GTT GTC TC-3′, ACTIN : forward, 5′-GCG AGA AGA TGA CCC AGA TC-3′, reverse, 5′-GGA TAG CAC AGC CTG GAT AG-3′.

### Plasmids and stable transfection

Dominant-negative p38 MAPK plasmid (pcDNA3/FLAG-p38 MAPK T180A/Y182F) and 670-1/MKK6EE plasmid were gifts from Dr. DS Min (Busan National University, Korea) and Dr. J Campisi (Buck Institute for Age Research, Novato, CA, USA), respectively. 3×10^5^ cells were seeded and incubated in 6-well plate until 70% confluent, and transfected with pcDNA3/FLAG-p38 MAPK T180A/Y182F using Lipofectamine 2000 (Invitrogen). The medium containing the plasmid and transfection reagent was replaced with a fresh one at 24 h post-transfection. The transfected cells were stabilized for 24 h, and then challenged with 1 mg/ml G418 until the cells became confluent. To generate lentiviral supernatants to infect H460 cells, HEK293T cells were transfected with a670-1/MKK6EE leniviral vector, the pHR' CMV 8.2 deltaR viral packaging plasmid, and the pCMV-VSV-G envelope plasmid (kindly provided by Dr. Bogoyevitch at Melbourne University, Australia) using Lipofectamine 2000. After 24 h, the medium was changed with fresh complete medium. Following additional incubation for 24-48 h, the viral supernatants were collected and challenged to H460 cells in complete medium containing 8 μl/ml polybrene (Sigma). The infected cells were stabilized for 24 h, and challenged with puromycin (2 μg/ml) treatment until the cells become confluent.

### Nuclear/cytosol extraction

To extract cytosol fraction, cell pellets were lysed with buffer A [10 mM HEPES (pH 7.9), 1.5 mM MgCl_2_, 10 mM KCl, 0.5 mM DTT, 1 mM EDTA, 0.5% NP-40, protease inhibitor cocktail, and phosphatase inhibitors (1 mM Na_3_VO_4_ and 100 mM NaF)] for 20 min on ice. The supernatant containing cytosolic proteins was collected by centrifugation (3,000 rpm, 10 min, 4°C). Then the pellet was washed with buffer A for three times, and lysed with buffer C [20 mM HEPES (pH 7.9), 1.5 mM MgCl_2_, 420 mM NaCl, 0.2 mM EDTA, 0.5 mM DTT, 25% glycerol, protease inhibitor cocktail, and phosphatase inhibitors (1 mM Na_3_VO_4_ and 100 mM NaF)] for 30 min on ice with vigorous vortexing every 10 min. The supernatant containing nuclear proteins was obtained by centrifugation (13,000 rpm, 20 min, 4°C). To check any cross-contamination between nuclear and cytosolic fractions, PARP and β-tubulin were used as markers for nuclear and cytosolic fractions, respectively.

### Gene silencing by siRNA transfection

3×10^5^ cells were seeded in a 6-well plate and simultaneously transfected with 100 pmoles of siRNA and 5 μl of Lipofectamine RNAiMAX Transfection Reagent (Invitrogen) according to the manufacturer's instructions. After 24 h, the medium was changed with fresh complete medium. The transfected cells were harvested at 48 h post-transfection for further analysis.

### Statistical analysis

Each result is expressed as the mean ± SD of data obtained from triplicate experiments. A statistical analysis was performed by Student's t-test. Differences less than *P* < 0.05 were considered statistically significant.

## SUPPLEMENTARY FIGURES


