# Supplementary material for: p38 MAPK-induced MDM2 degradation confers paclitaxel resistance through p53-mediated regulation of EGFR in human lung cancer cells
Source: Oncotarget. 2016 Jan 19;7(7):8184–99. doi: 10.18632/oncotarget.6945 (PMC4884985; doi:10.18632/oncotarget.6945)
Supplement: Supplementary file 1 [file oncotarget-07-8184-s001.pdf]

SUPPLEMENTARY FIGURES

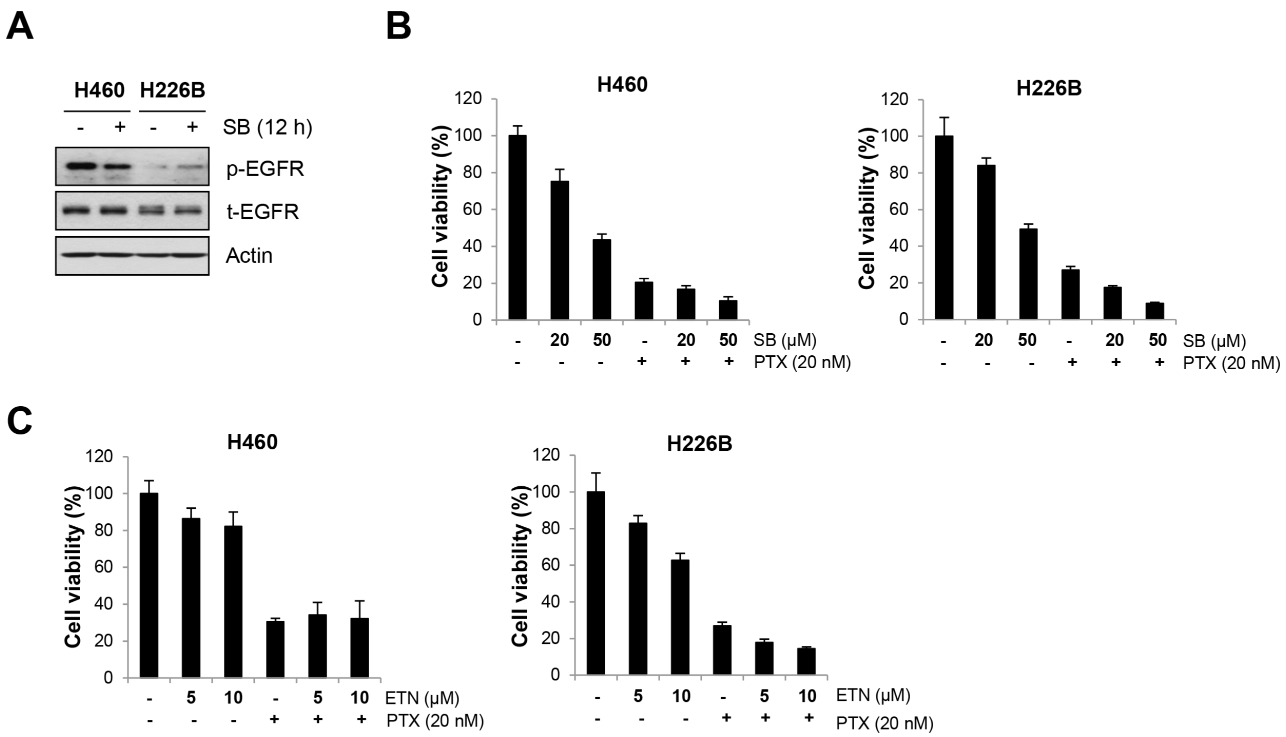

Supplementary Figure S1: p38 MAPK/EGFR axis is not functional in parental cells. A. H460 and H226B cells were incubated with SB203580 (SB; 50 μM) for 12 h. The expressions of p-EGFR and t-EGFR were analyzed using western blot analysis. Actin was used as an internal control. **B** and **C.** H460 and H226B cells were treated with (B) SB203580 (SB) or (C) erlotinib (ETN) in a dose-dependent manner in the presence of PTX (20 nM) for 72 h. Cell viability was determined by the MTT assay. The data are presented as the mean ± SD.

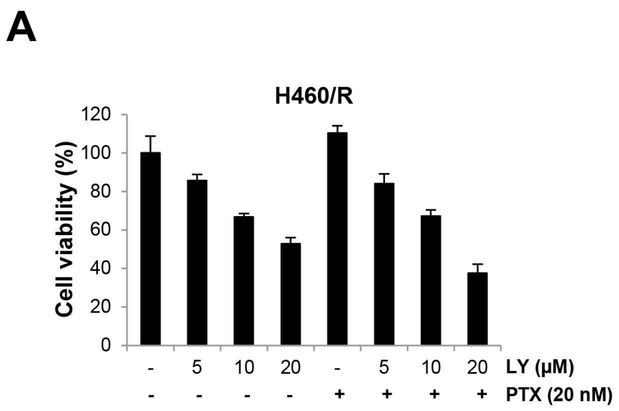

Supplementary Figure S2: AKT and JNK are not related with the generation of PTX resistance in H460/R cells. H460/R cells were treated with **A.** LY294002 (LY) or **B.** SP600125 (SP) in a dose-dependent manner in the presence of PTX (20 nM) for 72 h. Cell viability was determined by the MTT assay. The data are presented as the mean ± SD.

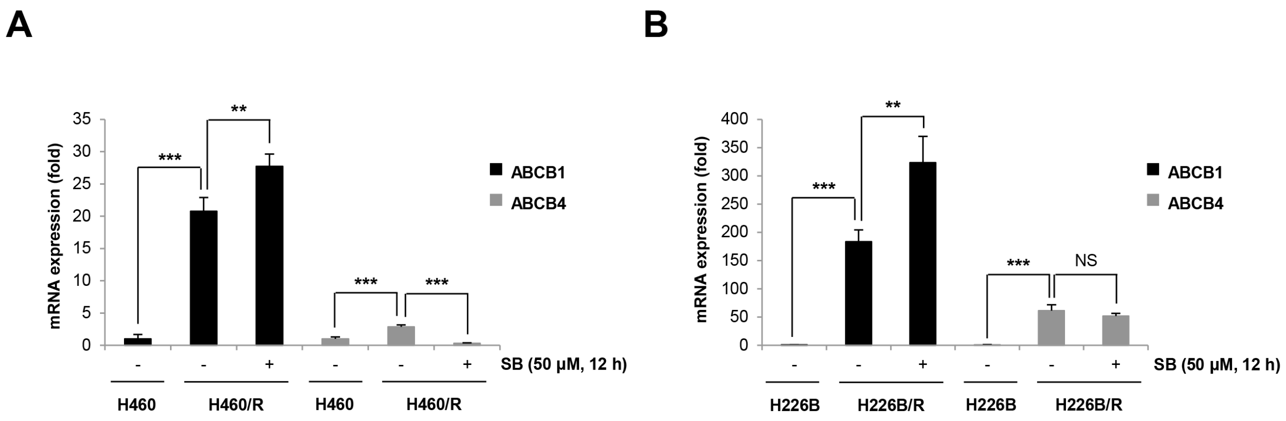

Supplementary Figure S3: p38 MAPK-conferred PTX resistance is independent on the regulation of MDR-related genes. The basal mRNA level of *ABCB1* and *ABCB4* in **A.** H460/R cells and **B.** H226B/R cells compared with their respective parental cells was investigated by real-time PCR. Cells treated with SB203580 (SB; 50 μM) for 12 h were also examined to quantify the mRNA expression of *ABCB1* and *ABCB4* using real-time PCR. The data are presented as the mean ± SD. The statistical significance was analyzed using Student's t-test (NS = not significant, ** $P < 0.01$, *** $P < 0.001$ vs. the respective control).
